# Formal and informal venous thromboembolism risk assessment and impact on prescribing of thromboprophylaxis: a retrospective cohort study

**DOI:** 10.1007/s11096-023-01578-w

**Published:** 2023-04-19

**Authors:** Megan Kemp, Amy Hai Yan Chan, Jeff Harrison, Hannah Rogers, Adele Zhao, Harleen Kaur, Genevieve Tang, Esther Yang, Kebede Beyene

**Affiliations:** 1 School of Pharmacy, Faculty of Medical and Health Sciences, The University of Auckland, Auckland, New Zealand; 2 Pharmacy Department, Auckland City Hospital, Auckland District Health Board, Auckland, New Zealand; 3 Department of Pharmaceutical and Administrative Sciences, St Louis College of Pharmacy, University of Health Sciences and Pharmacy in St. Louis, 1 Pharmacy Place, St. Louis, MO 63110 USA

**Keywords:** Thromboembolic prophylaxis, Venous thrombosis, Venous thromboembolism prophylaxis, VTE risk assessment

## Abstract

**Background:**

Hospital-acquired thrombosis (HAT) is a leading cause of preventable death and disability worldwide. HAT includes any venous thromboembolic (VTE) event occurring in-hospital or within 90-days of hospitalisation. Despite availability of evidence-based guidelines for HAT risk assessment and prophylaxis, guidelines are still underutilised.

**Aim:**

To determine the proportion of patients who developed HAT that could have been potentially prevented with appropriate VTE risk assessment and prophylaxis at a large public hospital in New Zealand. Additionally, the predictors of VTE risk assessment and thromboprophylaxis were examined.

**Method:**

VTE patients admitted under general medicine, reablement, general surgery, or orthopaedic surgery service were identified using ICD-10-AM codes. Data were collected on patient characteristics, VTE risk factors, and the thromboprophylaxis regimen prescribed. The hospital VTE guidelines were used to determine rates of VTE risk assessment and the appropriateness of thromboprophylaxis.

**Results:**

Of 1302 VTE patients, 213 HATs were identified. Of these, 116 (54%) received VTE risk assessment, and 98 (46%) received thromboprophylaxis. Patients who received VTE risk assessment were 15 times more likely to receive thromboprophylaxis (odds ratio [OR] = 15.4; 95% CI 7.65–30.98) and 2.8 times more likely to receive appropriate thromboprophylaxis (OR = 2.79; 95% CI 1.59–4.89).

**Conclusion:**

A large proportion of high-risk patients who were admitted to medical, general surgery and reablement services and who developed HAT did not receive VTE risk assessment and thromboprophylaxis during their index admission, demonstrating a significant gap between guideline recommendations and clinical practice. Implementing mandatory VTE risk assessment and adherence to guidelines to improve thromboprophylaxis prescription in hospitalised patients may help reduce the burden of HAT.

**Supplementary Information:**

The online version contains supplementary material available at 10.1007/s11096-023-01578-w.

## Impact statements

- Approximately half of all VTEs occur during or within 3 months after hospitalization. This represents a key window of opportunity to intervene to prevent VTEs.
- Despite the availability of evidence-based guidelines for VTE risk assessment and prophylaxis in hospitalized patients, adherence to guidelines is low.
- Our study found that patients receiving VTE risk assessment were 15 times more likely to get thromboprophylaxis, highlighting the importance of VTE assessment.
- Further research is needed to identify strategies to support VTE risk assessment and appropriate thromboprophylaxis and improve guideline adherence.

## Introduction

Hospital-acquired thrombosis (HAT) is one the leading causes of preventable death and disability worldwide. HAT is defined as any venous thromboembolic event (VTE, such as deep vein thrombosis [DVT] or pulmonary embolism [PE]) occurring during a hospital stay or within 90 days post-discharge [1]. Unrecognised and/or untreated VTE is associated with long-term risks causing significant healthcare and economic burden [2]. Inpatients have a ten-fold increased risk of developing VTE [3] and up to three-quarters of VTE-related deaths are associated with hospitalization [1, 4–10].

Prevention is key to minimising HAT and involves identifying at-risk patients and appropriately prescribing thromboprophylaxis during patients’ index admission. VTE risk assessment tools (e.g. the Padua Prediction Score and Caprini VTE score) have proven to be a safe and cost-effective method to identify high-risk patients requiring thromboprophylaxis and low-risk patients who do not [11, 12]. A significant reduction in VTE events and HAT deaths within 90 days post-discharge was observed in hospitals achieving > 90% VTE risk assessment [13–15]. However, despite supporting evidence, VTE guidelines are still underutilized [16]. International literature reports that only 16–33% of medical patients at risk of VTE receive thromboprophylaxis compared to 90% of at-risk surgical patients [13].

Risk factors for HAT are multifactorial. Approximately 80% of HAT patients have one or more risk factors for VTE [17, 18], including older age, multiple comorbidities, obesity, personal or family VTE history, immobilisation, surgery, cancer, chemotherapy, trauma, and longer hospital stays [2, 18–20]. Some of these risk factors are modifiable (e.g. obesity, immobilisation), while others, like advancing age, comorbidities, and genetic factors, are not. Studies also reported several reasons for clinicians' lack of guideline compliance, including concern over risk of bleeding, underestimation of VTE risk, and lack of consensus on the benefits versus risks of prophylaxis [1, 2, 21].

Previous studies are mostly from the US and focus on determining the incidence and risk factors for VTE [13, 22]. There is limited information about the predictors of VTE risk assessment and thromboprophylaxis during hospital admission, which provides a unique opportunity to intervene to improve VTE prophylaxis during inpatient stays. Our study addresses this gap.

### Aim

This study aimed to determine the proportion of patients who developed HAT across four services (wards) at a large public hospital that could have been potentially prevented with appropriate VTE risk assessment and prophylaxis. The predictors of VTE risk assessment and prescription of appropriate thromboprophylaxis were also examined.

### Ethics approval

This study was approved by the Auckland Health Research Ethics Committee (Ref No. AH1323; date: May 06, 2020).

## Method

### Study design and setting

This was a hospital-based retrospective cohort study using electronic medical records at a large public hospital in New Zealand (NZ).

### Inclusion/exclusion criteria

The study cohort included adult patients (≥ 18 years) admitted to general medicine, reablement, general surgery, and orthopaedic services at a large public hospital between 1 January 2015 and 31 December 2019. Only patients with valid electronic medical records were included. Patients with HAT-diagnosis referred to the hospital from private practices were excluded as data were not accessible. Patients admitted to any other than the services mentioned above were also excluded. Patients with direct admission to critical care or spinal surgery were excluded as they have significantly different risk factors and standard VTE risk assessment models do not always apply [23]. Patients with superficial vein (e.g., cephalic and basilic vein thrombosis) were also excluded, primarily for comparability of results to literature. All included VTE events required confirmation by computed tomography pulmonary angiogram (CTPA) or Doppler ultrasound or be a likely cause of death as confirmed in clinical notes; any probable cases, (thrombo)phlebitis or non-VTE events were excluded.

### Data collection

Data on eligible patients during the study period were obtained from the Business Intelligence Unit of the study hospital. ICD-10 AM codes (I26.xx, I80.xx, I81, I82.xx, O082, O223, O871, O882) were used to identify patients with VTE (see Supplementary file 1) [24]. Identified patients were compared against discharge summaries to confirm the diagnosis and assigned ICD-10-AM codes. Each patient was assessed for the completion of VTE risk assessment and thromboprophylaxis appropriateness. The data were extracted by five research team members (AZ, HK, MK, GT, EY). All data collectors had clinical training and attended a data collection training run by hospital collaborators. Some trial cases were selected to demonstrate the data collection process to ensure all data collectors followed the same method. After the completion of data collectors training, pilot data collection was conducted to ensure a consistent data collection process. During piloting, two cases were assigned to each data collector, who worked independently and recorded data on Excel templates with predetermined variables of interest. These cases were then rotated between the other data collectors to ensure uniformity in the method. The pilot data were reviewed again by senior clinical research team members to check for any incorrect and missing data, and errors were subsequently remediated. The 5 research team members all contributed equally to the data collection process. All the research team members had regular weekly meetings to ensure the quality of the data collection process. 3 M ChartView software was used to access electronic patient files for eligibility screening and data collection.

### Risk assessment and appropriateness of thromboprophylaxis

The hospital thromboprophylaxis guideline (see Supplementary file 2) was used to assess VTE risk and to determine whether patients received appropriate thromboprophylaxis. When assessing thromboprophylaxis appropriateness, the chosen prophylaxis was compared against the recommended agent and dose (if pharmacological) for that patient’s risk stratification. Thromboprophylaxis contraindications were taken into consideration. Following the hospital medical risk assessments flow-chart (see Supplementary file 3), the first criterion was whether ‘immobilisation was expected within 72 h (including prior to admission)’. Medical and reablement patients who received ‘no prophylaxis’ were ultimately noted with ‘appropriate prophylaxis’ if immobilisation was not expected for at least 72 h (including prior to admission) regardless of individual patient risk factors. Orthopaedic and surgical patients who received ‘no prophylaxis’ were recorded as ‘appropriate prophylaxis’ if they had significant contraindications (e.g. active bleeding, recent upper GI ulcer, cellulitis) documented for pharmacological and/or mechanical prophylaxis as determined from the clinical notes.

### Predictors of VTE risk assessment and prophylaxis

Potential risk factors for VTE risk assessment and prophylaxis were collected based on existing literature. Socio-demographic data were collected, including age at index hospital admission, sex, height, weight, ethnicity, and socioeconomic deprivation index. Additionally, patient-related risk factors were collected, including previous VTE history, restricted mobility, current pregnancy, trauma, and (severe) infection requiring IV antibiotics. Limited mobility was defined as inability to walk unassisted, wheelchair or bed bound. Other risk factors included alcohol consumption, smoking status, serum creatinine level, and admission to intensive care unit (ICU) during hospital stay. Comorbidities recorded included recent ischaemic stroke, myocardial infarction (MI), heart failure (HF), chronic obstructive pulmonary disease (COPD), inflammatory bowel disease (IBD), active cancer, diabetes, and thrombophilia. Stroke, MI and HF were limited to events occurring within 6-months before the index hospital admission as literature indicated that recent events were associated with the highest risk. High-risk medications, including hormone replacement therapy (HRT), oral contraceptive pill (OCP), antidepressants, and chemotherapy, within 6-months before the index hospital admission were documented. Additionally, data relating to the index hospital admission, such as date of admission, service, primary diagnosis, secondary diagnosis, length of stay (LOS), procedure length (if applicable), completion of VTE risk assessment (formal, informal, or none), VTE prophylaxis including date initiated, type (pharmacological or non-pharmacologic), dose (if pharmacological), and duration were collected. The presence of contraindications/precautions for thromboprophylaxis was documented.

### Statistical analysis

Data were analysed using SPSS version 27. Descriptive statistics were used to summarise the baseline characteristics of patients. The proportion of patients who received thromboprophylaxis and those who did not was determined. Multivariable logistic regression models were used to determine predictors of outcome measures. The outcome measures were receiving VTE risk assessment, receiving thromboprophylaxis, and receiving appropriate thromboprophylaxis. The logistic regression models included the following potential risk factors: sex, age, ethnicity, socioeconomic deprivation index, smoking status, service type, LOS, comorbidities, and concurrent medications. Adjusted odds ratios (AORs) and 95% confidence intervals (CIs) were calculated. We also conducted an exploratory analysis to evaluate the impact of formal or informal VTE risk assessment on thromboprophylaxis, using bivariate logistic regression models. All tests were considered statistically significant at *p* < 0.05.

## Results

**Fig. 1:**
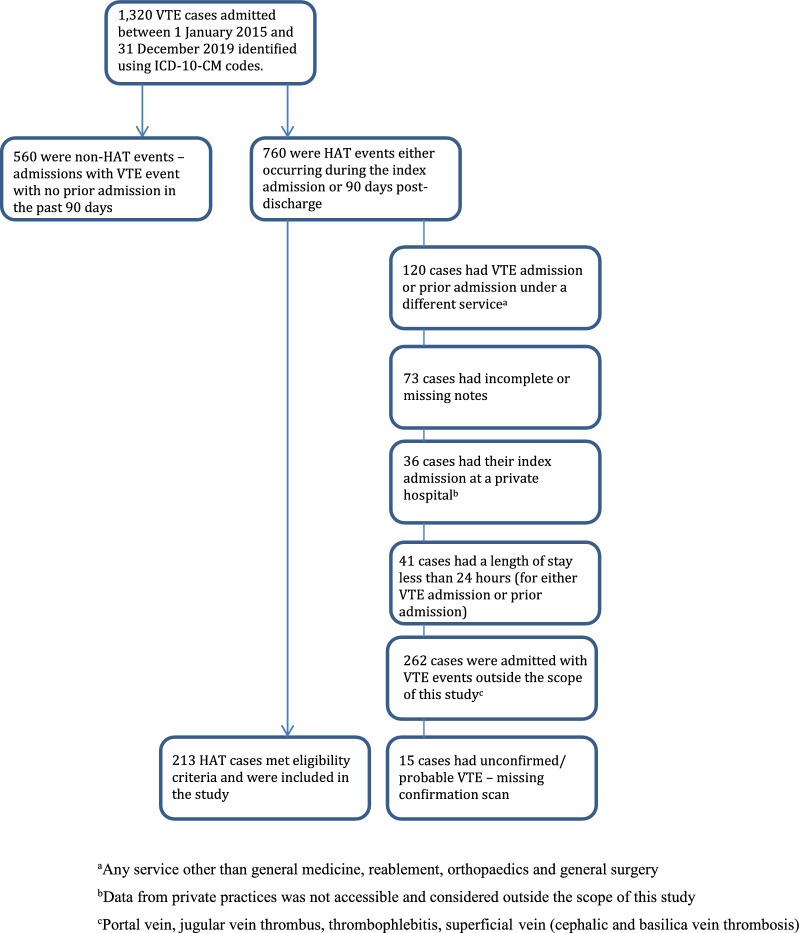
Patient selection flow chart

A total of 1302 VTE cases were admitted to the hospital during the study period; of these, 213 patients were eligible for inclusion in the final data analysis (see Fig. 1). Table 1 shows the characteristics of the study sample; primarily NZ-European (160/213; 75%), female (129/213; 60.6%), and ≥ 65 years of age (152/213; 71.4%). Two-thirds of the patients were admitted to either medical or reablement service, and their median LOS was 12.3 days. Nearly half (104/213; 48.8%) of the patients presented with DVT, and 46.9% (100/213) presented with PE. Over half of the patients (116/213; 54.5%) received some form of VTE risk assessment. Orthopaedic (38/50; 76%) and reablement (41/74; 55.4%) services had the highest rates of VTE risk assessment. Only a small proportion (32/213; 15%) of patients received formal VTE risk assessment, whereas 43.7% (93/213) of the overall sample received informal risk assessment. Formal risk assessment was relatively higher in orthopaedic patients (20/50; 40%) compared to patients admitted to general surgery (3/24; 12.5%), medical (7/65; 10.8%) or reablement (2/74; 2.2%) services. Regarding thromboprophylaxis, 46.2% (98/213) of patients received any form of thromboprophylaxis, and 56.8% (121/213) received appropriate thromboprophylaxis. General surgery had the highest rates of providing prophylaxis (20/24; 83.3%) and of providing appropriate prophylaxis (19/24; 79%). Medical service gave thromboprophylaxis less frequently (16/65; 24.6%) than other services (see Table 1).

**Table 1:** Characteristics of patients diagnosed with hospital-acquired thrombosis and proportion of patients who received VTE risk assessment and thromboprophylaxis by service type

	Medical N (%) = 65 (30.5)	Reablement N (%) = 74 (34.7)	General surgery N (%) = 24 (11.3)	Orthopaedics N (%) = 50 (23.5)	Total (N%) = 213 (100)
Sex					
Male	26 (40)	27 (36.5)	12 (50)	19 (38)	84 (39.4)
Female	39 (60)	47 (63.5)	12 (50)	31 (62)	129 (60.6)
Age					
< 65 years	28 (43.1)	6 (8.1)	11 (45.8)	16 (32)	61 (28.6)
65–84 years	29 (44.6)	42 (56.8)	13 (54.2)	31 (62)	115 (54)
≥ 85 years	8 (12.3)	26 (35.1)	0	3 (6)	37 (17.4)
Ethnicity					
NZ European	47 (72.3)	60 (81.1)	19 (79.2)	34 (68)	160 (75.1)
Māori	6 (9.2)	2 (2.7)	1 (4.2)	3 (6)	12 (5.6)
Pacific peoples	7 (10.8)	4 (5.4)	1 (4.2)	6 (12)	18 (8.5)
Asian	5 (7.7)	8 (10.8)	2 (8.3)	6 (12)	21 (9.9)
Other	0	0	1 (4.2)	1 (2)	2 (0.9)
Deprivation Index^a^					
Quintile 1	11 (16.9)	21 (28.4)	0	8 (16)	40 (18.8)
Quintile 2	12 (18.5)	21 (28.4)	5 (20.8)	12 (24)	50 (23.5)
Quintile 3	20 (30.8)	22 (29.7)	11 (45.8)	12 (24)	65 (30.5)
Quintile 4	8 (12.3)	1 (1.4)	4 (16.7)	6 (12)	19 (8.9)
Quintile 5	14 (21.5)	9 (12.2)	4 (16.7)	12 (24)	39 (18.3)
Length of hospital stay					
≤ 10 days	39 (60)	10 (13.5)	13 (54.2)	33 (66)	95 (44.6)
> 10 days	26 (40)	64 (86.5)	11 (45.8)	17 (34)	118 (55.4)
Type of VTE event					
DVT	28 (43.1)	37 (50)	11 (45.8)	28 (56)	104 (48.8)
PE	33 (50.8)	32 (43.2)	13 (54.2)	22 (44)	100 (46.9)
Both DVT and PE	4 (6.2)	5 (6.8)	0	0	9 (4.2)
Received any form of VTE risk assessment	24 (36.9)	41 (55.4)	13 (54.2)	38 (76)	116 (54.5)
Received formal VTE Risk assessment	7 (10.8)	2 (2.7)	3 (12.5)	20 (40)	32 (15)
Received informal VTE risk assessment	20 (30.8)	39 (52.7)	11 (45.8)	23 (46)	93 (43.7)
Received both formal and Informal VTE risk assessment	3 (4.6)	0	1 (4.2)	5 (10)	9 (4.2)
Received any thromboprophylaxis	16 (25)	30 (40.5)	20 (83.3)	32 (64)	98 (46.2)
Received appropriate thromboprophylaxis	34 (52.3)	41 (55.4)	19 (79.2)	27 (54)	121 (56.8)
Type of prophylaxis prescribed					
Pharmacological	13 (20)	20 (27)	7 (29.2)	23 (46)	63 (29.6)
Non-pharmacological	0	2 (2.7)	2 (8.3)	0	4 (1.9)
Both pharmacological and non-pharmacological	3 (4.6)	8 (10.8)	8 (33.3)	11 (22)	30 (14.1)

^a^Quintile 1: decile 1 and 2; Quintile 2: decile 3 and 4; Quintile 3: decile 5 and 6; Quintile 4: decile 7 and 8; Quintile 5: decile 9 and 10

Most patients had ≥ 1 known risk factor for VTE. The most prevalent risk factor was immobility (115/213; 54%), followed by trauma (96/213; 45%), and serious infection requiring IV antibiotics (85/213; 39.9%). Only a small proportion (38/213; 17.8%) of patients were taking medications associated with increased risk of HAT, most commonly antidepressants (19/213; 8.9%) and cancer medications (14/213; 6.6%). Over half (113/213; 53.1%) of the patients had no comorbidities associated with increased risk of HAT, but 15% (32/213) had active cancer (see Table 2).

**Table 2:** Distribution of risk factors for hospital-acquired thrombosis by service type (N = 213)

	Medical N (%) = 65 (30.5)	Reablement N (%) = 74 (34.7)	General surgery N (%) = 24 (11.3)	Orthopaedic N (%) = 50 (23.5)	Total N (%) = 213 (100)
Risk factors					
History of VTE^a^	11 (16.9)	3 (4.1)	4 (16.7)	8 (16)	26 (12.2)
Current pregnancy	0	0	0	1 (2)	1 (0.5)
Current smoker	7 (10.8)	2 (2.7)	5 (20.8)	6 (12)	20 (9.4)
Ex-smoker^b^	12 (18.5)	14 (18.9)	1 (4.2)	12 (24)	39 (18.3)
Trauma	26 (40)	37 (50)	8 (33.3)	25 (50)	96 (45)
Serious infection	28 (43.1)	27 (36.5)	8 (33.3)	22 (44)	85 (39.9)
ICU stay^c^	0	3 (4.1)	0	3 (6)	6 (2.8)
Restricted mobility	38 (59.4)	43 (58.1)	10 (41.7)	24 (48)	115 (54.2)
Comorbidities					
Stroke^d^	3 (4.6)	5 (6.8)	0	2 (4)	10 (4.7)
Myocardial infarction^d^	10 (15.4)	6 (8.1)	2 (8.3)	7 (14)	25 (11.7)
CHF^e^	4 (6.2)	8 (10.8)	3 (12.5)	2 (4)	17 (8)
COPD^f^	6 (9.2)	4 (5.4)	4 (16.7)	7 (14)	21 (9.9)
Active cancer	7 (10.8)	10 (13.5)	6 (25)	9 (18)	32 (15)
Thrombophilia	0	1 (1.4)	1 (4.2)	0	2 (0.9)
Irritable bowel disease	2 (3.1)	0	0	0	2 (0.9)
Diabetes	15 (23.1)	9 (12.2)	2 (8.3)	4 (8)	30 (14.1)
Any comorbidity	36 (55.4)	30 (40.5)	13 (54.2)	21 (42)	100 (46.9)
Risk associated medications					
Oral contraceptive pill	4 (6.2)	0	0	0	4 (1.9)
HRT^g^	1 (1.5)	0	0	1 (2)	2 (0.9)
Antidepressant	5 (7.7)	7 (9.5)	3 (12.5)	4 (8)	19 (8.9)
Chemotherapy	3 (4.6)	2 (2.7)	4 (16.7)	5 (10)	14 (6.6)
Any medication	12 (18.5)	9 (12.2)	7 (29.2)	10 (20)	38 (17.8)

^a^Venous thromboembolism

^b^Smoke free in the last 28 days

^c^ICU = Intensive Care Unit

^d^Event occurring within 6 months before index admission

^e^Congestive heart failure

^f^Chronic obstructive pulmonary disease

^g^Hormone replacement therapy

In multivariable logistic regression analysis, the odds of receiving VTE risk assessment were 3.7 times higher among patients with non-European ethnicity than NZ-Europeans (AOR = 3.72; 95% CI 1.63–8.45). The odds of receiving VTE risk assessment were 5.3 times higher among orthopaedic and surgical patients compared to medical patients (AOR = 5.3; 95% CI 2.39–11.83). Conversely, patients with ≥ 2 comorbidities had lower odds of receiving VTE risk assessment (AOR = 0.32; 95% CI 0.12–0.86). Patients living in areas with socioeconomic deprivation quintile-2 (AOR = 0.22; 95% CI 0.08–0.61), quintile-3 (AOR = 0.34; 95% CI 0.13–0.88), and quintile-5 (AOR = 0.29; 95% CI 0.10–0.90) had lower odds of receiving VTE risk assessment compared to those living in the least deprived areas (quintile-1) (see Table 3).

**Table 3:** Multivariable logistic regression examining predictors of VTE risk assessment (N = 213)

	Adjusted odds ratio (95% CI)	*p* value
Sex		
Female (reference)	1	
Male	0.92 (0.48–1.79)	0.812
Ethnicity		
NZ European (reference)	1	
Other	**3.72 (1.63–8.46)**	**0.002**
Age		
< 65 years (reference)	1	
65–84 years	0.63 (0.29–1.36)	0.237
≥ 85 years	0.79 (0.27–2.33)	0.669
Deprivation index^a^		
Quintile 1 (reference)	1	
Quintile 2	**0.22 (0.08–0.61)**	**0.003**
Quintile 3	**0.34 (0.13–0.88)**	**0.027**
Quintile 4	0.36 (0.36–1.37)	0.133
Quintile 5	**0.29 (0.10–0.89)**	**0.031**
Length of hospital stay		
≤ 10 days (reference)	1	
> 10 days	1.83 (0.90–3.73)	0.097
Service type		
Medical (reference)	1	
Orthopaedic/surgery	**5.32 (2.39–11.83)**	** < 0.001**
Reablement	1.86 (0.76–4.52)	0.174
Number of comorbidities		
None (reference)	1	
1	0.57 (0.28–1.14)	0.112
≥ 2	**0.32 (0.12–0.86)**	**0.024**
Any concurrent medicines		
No (reference)	1	
Yes	0.62 (0.27–1.41)	0.250
Trauma		
No (reference)	1	
Yes	0.77 (0.39–1.52)	0.455
Intravenous antibiotics		
No	1	
Yes	0.80 (0.41–1.57)	0.518
Restricted mobility		
No (reference)	1	
Yes	0.89 (0.45–1.79)	0.747

^a^Quintile 1: decile 1 and 2

Quintile 2: decile 3 and 4

Quintile 3: decile 5 and 6

Quintile 4: decile 7 and 8

Quintile 5: decile 9 and 10

Bold text indicates a significant statistical association

The odds of receiving thromboprophylaxis were 7.3 times higher among orthopaedic and surgical patients than medical patients (AOR = 7.3; 95% CI 3.15–16.78). Patients with a hospital stay ≥ 10 days had 2.2 times greater odds of receiving thromboprophylaxis than those with < 10 days of hospital stay (AOR = 2.16; 95% CI 1.01–4.63). Patients with ≥ 2 comorbidities were less likely to receive thromboprophylaxis (AOR = 0.31; 95% CI 0.11–0.86). No other significant predictors for thromboprophylaxis were found (see Table 4).

**Table 4:** Multivariate logistic regression examining predictors of thromboprophylaxis administration (N = 202)

	Adjusted Odds ratio (95% CI)	*p* value
Sex		
Female (reference)	1	
Male	1.52 (0.76–3.05)	0.238
Ethnicity		
NZ European (reference)	1	
Other	1.57 (0.72–3.41)	0.253
Age		
< 65 years (reference)	1	
≥ 65 years	0.62 (0.29–1.34)	0.227
NZDep2013 index^a^		
Low deprivation (reference)	1	
Moderate deprivation	1.22 (0.58–2.56)	0.609
High deprivation	1.30 (0.49–3.44)	0.602
Smoking status		
Non-smoker (reference)	1	
Current/ex-smoker	1.02 (0.49–2.14)	0.955
Length of hospital stay		
≤ 10 days (reference)	1	
> 10 days	**2.16 (1.01–4.63)**	**0.047**
Service type		
Medical (reference)	1	
Orthopaedic/surgery	**7.28 (3.15–16.78)**	** < 0.001**
Reablement	1.58 (0.63–4.01)	0.333
Number of comorbidities		
None (reference)	1	
1	0.60 (0.29–1.23)	0.163
≥ 2	**0.31 (0.11–0.86)**	**0.024**
Number of concurrent medicines		
No (reference)	1	
≥ 1	0.75 (0.31–1.78)	0.511
Trauma (reference = No)		
Yes	0.99 (0.49–1.97)	0.966
Intravenous antibiotics (reference = No)		
Yes	1.91 (0.94–3.85)	0.072
Restricted mobility (reference = No)		
Yes	0.67 (0.33–1.34)	0.254

^a^Low deprivation = decile 1–3; moderate deprivation = decile 4–7; high deprivation = decile 8–10

Bold text indicates a significant statistical association

In bivariate logistic regression, the odds of receiving any thromboprophylaxis were 8.3 times higher for patients who received formal VTE risk assessment than those who did not (OR = 8.29; 95% CI 3.05–22.53). Likewise, patients who received informal VTE risk assessment were 6.6 times more likely to receive any thromboprophylaxis (OR = 6.64; 95% CI 3.63–12.15) and 2.9 times more likely to receive appropriate thromboprophylaxis (OR = 2.88; 95% CI 1.62–5.12). Additionally, patients who received either formal or informal VTE risk assessment were 15.4 times more likely to receive any thromboprophylaxis (OR = 15.37; 95% CI 7.65–30.98) and 2.8 times more likely to receive appropriate thromboprophylaxis (OR = 2.79; 95% CI 1.59–4.89) (see Table 5).

**Table 5:** Bivariate logistic regression models examining the association between VTE risk assessment and receiving thromboprophylaxis for all services (N = 213)

	Receiving any thromboprophylaxis		Receiving appropriate thromboprophylaxis	
	Odd ratio (95% CI)	*p* value	Odd ratio (95% CI)	*p* value
Formal VTE Risk assessment				
No	1		1	
Yes	**8.29 (3.05, 22.53)**	** < 0.001**	1.54 (0.70, 3.39)	0.277
Informal VTE risk assessment				
No	1		1	
Yes	**6.64 (3.63, 12.15)**	** < 0.001**	**2.88 (1.62, 5.12)**	** < 0.001**
Formal or Informal VTE risk assessment				
No	1		1	
Yes	**15.37 (7.65, 30.89)**	** < 0.001**	**2.79 (1.59, 4.89)**	** < 0.001**

Bold text indicates a significant statistical association

## Discussion

### Statement of key findings

This is one of a few studies that examined formal and non-formal VTE risk assessment, pharmacologic and non-pharmacologic prophylaxis as well as predictors of VTE risk assessment in hospitalised patients. We also compared the rates of risk assessment and prophylaxis across different services, and the inclusion of older people services (i.e. reablement) makes our study unique since most previous studies only focused on medical and surgical patients. Of the 213 patients identified with HAT, 54.5% (116/213) received some form of risk assessment during their index admission; however, only 15% (32/213) were formally assessed. Ninety-eight patients (46.2%) received any thromboprophylaxis during their index admission, and 56.8% (121/213) received appropriate thromboprophylaxis. These findings are in-line with other studies which have demonstrated low rates of risk assessment and thromboprophylaxis in hospitalised patients [2, 25].

### Strengths and weaknesses

This study has some limitations. Patient information collected was dependent on the accuracy of the documentation during the index hospital admission. For example, mechanical prophylaxis was poorly documented and may have been significantly under-estimated. Our data are from one hospital and may not be generalisable to the rest of NZ. The small proportion of patients from individual ethnic groups made it difficult to draw definitive conclusions through sub-group analysis. HAT events were identified from a single hospital; we might have missed HAT cases which presented to other institutions. In addition, this study used the Hospital VTE guidelines to assess patients’ VTE risk and determine thromboprophylaxis appropriateness. In contrast, many other studies utilised the ACCP guidelines. Thus, our results may not be directly comparable with other studies.

Despite the above limitations, our findings can help clinicians and policy makers to design strategies to minimise the risk of HAT. Since 2010, the National Health Service (NHS) has made VTE risk assessment a key performance indicator with a target of assessing 95% of adult hospital admissions in England [26]. NZ and other countries may adopt similar approach to reduce morbidity and mortality associated with HAT.

### Interpretation

There is substantial research evidence supporting the place of risk assessment and thromboprophylaxis in surgical settings [1, 2, 21] than in non-surgical settings [18, 27, 28]. This may explain the higher rates of VTE risk assessment observed in surgical patients compared to medical patients in this study. Furthermore, our study showed orthopaedic clinicians were more likely to use informal risk assessment, which differs from the official Hospital-guidelines, likely due to the lack of specificity allowing for clinician discretion and preference based on experience. This is supported by literature, which found clinicians preferred to use their own clinical experience when prescribing thromboprophylaxis rather than adhering to VTE-guidelines [23].

Reablement contributed the largest number of cases to the study population. This service consists of four inpatient wards that deliver high-quality health care for older people and rehabilitation services for adults aged over 16 years. Thus, our results would provide relevant information to other institutions with similar services. The absence of a reablement-specific VTE-guideline and the complexity of these patients may explain the low rates of formal risk assessment.

In line with a previous study [28], in our study patients who received formal, informal or any form of risk assessment were more likely to be prescribed prophylaxis. Interestingly, those who received informal VTE risk assessment were 6.6 times more likely to receive any prophylaxis and 2.9 times more likely to receive appropriate prophylaxis. These findings suggest that other methods of risk assessment have superseded the current hospital guidelines, likely due to convenience and practicality. Further research is needed to assess the accuracy and safety of informal risk assessment and subsequent thromboprophylaxis prescription.

Surgical services had higher rates of prescribing any form of thromboprophylaxis, again likely due to the large amount of supporting evidence for thromboprophylaxis in surgical patients [1, 2, 21]. Reablement, compared to medical patients, had a high proportion of patients receiving any form (30/74; 40.5%) and appropriate thromboprophylaxis (41/74; 55.4%). Each reablement patient is transferred from a different ward, thus many of these patients would meet the first criteria of the medical flow chart, likely increasing the rate of thromboprophylaxis prescription compared to medical patients.

Data on the association between ethnicity/race and VTE are rare outside of North America. In US studies, African Americans had 30–60% higher incidence of VTE compared to White Americans, while other ethnic minorities (e.g., Asians, Pacific Islanders, and Hispanics) had lower risk of VTE [29, 30]. Due to the small sample size, we could not compare VTE risk assessment and prophylaxis between each ethnic group. However, we found that non-Europeans were 3.7 times more likely to receive VTE risk assessment compared to NZ Europeans. However, about 75% of our study participants were of NZ European ethnicity, and the lack of variation in our sample could affect the generalizability of our findings to non-European ethnicities. Apart from ethnicity, male sex, obesity, older age, a history of VTE, and immobility are well-established risk factors for VTE [31–33]. Thus, VTE risk assessment should consider these factors to ensure appropriate thromboprophylaxis of hospitalised patients.

Patients living in more socioeconomically deprived areas are likely to have reduced health literacy and poorer access and engagement with healthcare services [34]. In this study, these patients were found to be less likely to receive VTE risk assessment. This could lead to increased incidence of VTE, poorer health outcomes and mortality [34]. To bridge disparities between socioeconomic groups it is crucial for patients living in more deprived areas to receive risk assessment and appropriate thromboprophylaxis.

Patients with ≥ 2 comorbidities had lower odds of receiving risk assessment. This contradicts our hypothesis where more comorbid patients with increased risk of polypharmacy, treatment adverse effects and bleeding, would be more likely to receive risk assessment [35]. However, this could be due to the complexity of these patients, leading to difficulty in risk assessment and thromboprophylaxis prescription [1].

### Future research

Further research is needed at other hospitals (e.g. private, rural hospitals) to assess their respective VTE prevention protocols. It is evident that there is a lack of literature regarding the rates of VTE risk assessment by clinicians. More research in this area would be beneficial to better understand and address potential barriers and attitudes associated with incompletion of VTE risk assessment. Further prospective studies are also needed to assess the impact of VTE risk assessment and appropriate thromboprophylaxis on the subsequent development of HAT.

## Conclusion

A large proportion of high-risk patients who developed HAT did not receive VTE risk assessment and thromboprophylaxis during their index admission, demonstrating a significant gap between guideline recommendations and routine clinical practice. Implementation of mandatory VTE risk assessment and adherence to guidelines to improve thromboprophylaxis prescription in hospitalised patients may help in reducing mortality, morbidity and the economic burden associated with HAT. Further, clinicians need to pay attention to those who live in lower socioeconomic areas to reduce potential disparities in thromboprophylaxis and associated outcomes.

## Supplementary Information

Below is the link to the electronic supplementary material.

Supplementary file1 (DOCX 728 KB)
