# Supplementary material for: Formal and informal venous thromboembolism risk assessment and impact on prescribing of thromboprophylaxis: a retrospective cohort study
Source: Int J Clin Pharm. 2023 Apr 19;45(4):864–74. doi: 10.1007/s11096-023-01578-w (PMC10366250; doi:10.1007/s11096-023-01578-w)
Supplement: Supplementary file 1 — Supplementary file1 (DOCX 728 KB) [file 11096_2023_1578_MOESM1_ESM.docx]

# Supplementary file 1: ICD-10-AM codes for VTE

| I260 Pulmonary embolism with mention of acute cor pulmonale |
| --- |
| I269 Pulmonary embolism without mention of acute cor pulmonale |
| I801 Phlebitis and thrombophlebitis of femoral vein |
| I802 Phlebitis and thrombophlebitis of other deep vessels of lower extremities |
| I803 Phlebitis and thrombophlebitis of lower extremities, unspecific |
| I808 Phlebitis and thrombophlebitis of other sites |
| I809 Phlebitis and thrombophlebitis of unspecified site |
| I81 Portal vein thrombosis |
| I820 Budd-Chiari syndrome |
| I821 Thrombophlebitis migrans |
| I822 Embolism and thrombosis of vena cava |
| I823 Embolism and thrombosis of renal vein |
| I828 Embolism and thrombosis of other specified veins |
| I829 Embolism and thrombosis of unspecified vein |
| O082 Embolism following abortion and ectopic and molar pregnancy |
| O223 Deep phlebothrombosis in pregnancy |
| O871 Deep phlebothrombosis in the puerperium |
| O882 Obstetric blood-clot embolism |

**Supplementary file 2:** Adapted from Te Toka Tumai Auckland City Hospital VTE risk assessment guidelines

| **Risk category for medical/reablement patients** | **Risk category for surgical patients** | **Risk category for orthopaedic patients** |
| --- | --- | --- |
| **High**   - Immobilization anticipated for at least 72 hours (including prior admission)   AND one of the followings:   - History of DVT or PE - Active malignancy - Inflammatory bowel disease - AND at least 2 risk factors*** | **High**   - Orthopaedic surgery for hip or pelvic fracture or hip/knee arthroplasty - Multiple trauma - Major surgery and thrombophilia, active malignancy, or history of VTE - Major surgery and 3 or more VTE risk factors | **High**  Patients for elective total hip and total knee replacement, other elective surgery or proximal femoral fractures with at least one risk factor present (refer to surgical risk factors***) |
| **Moderate**   - Immobilization anticipated for at least 72 hours (including prior admission)   AND one of the following:   - History of DVT or PE - Active malignancy - Inflammatory bowel disease - OR at least 2 risk factors*** | **Moderate**   - Major surgery and less than three or more risk factors** - Minor surgery and thrombophilia, active malignancy or history of VTE - Minor surgery and less than three or more risk factors** | **Moderate**   - Patients for elective total hip and total knee replacement with at least one risk factor present (refer to surgical risk factors***) - Patients admitted with fracture/other injuries requiring a period of bed rest prior to definitive surgery |
| **Low**  Immobilisation anticipated for less than 72 hours | **Low**  Minor surgery and less than three risk factors | **Low**  Patients for elective total hip and total knee replacement with anticipated early mobilisation (day one) |

** Risk factors for surgical VTE risk assessment

- Age > 40
- Obesity(BMI>30)
- Mod-severe heart failure
- Severe airways disease
- HRT or oral contraceptive use
- Recent pregnancy
- Varicose veins
- Infection or inflammation
- Lower limb trauma
- Immobility

***Risk factors for medical VTE risk assessment

- Uncontrolled heart failure
- Chronic respiratory disease
- Acute rheumatological illness
- Severe infection
- Ischemic stroke
- Thrombophilia
- Myocardial ischemia/infarction
- BMI>30
- Recent surgery or trauma
- Oral contraceptives/HRT Prolonged immobility (>7 days)
- Age>60 years

# Supplementary file 3. Te Toka Tumai Auckland Hospital formal VTE risk assessment forms for general medicine, general surgery and on ADHB medicine charts


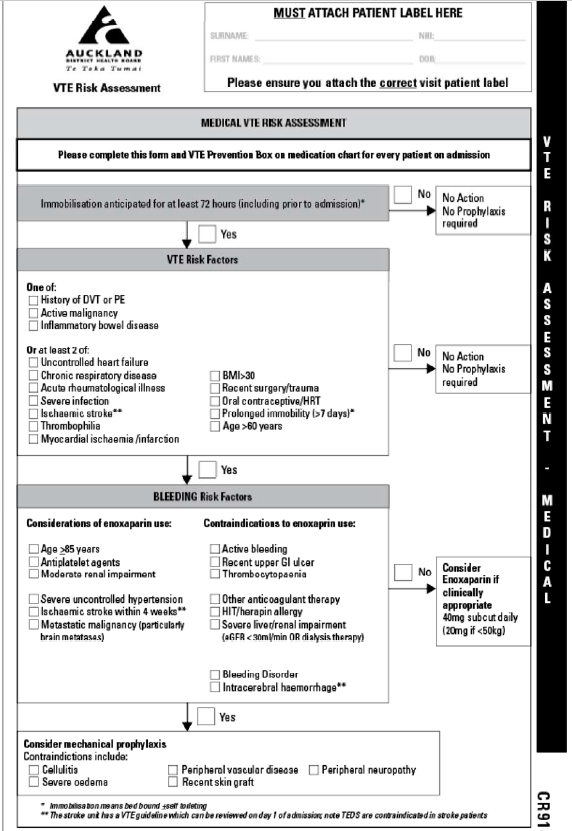


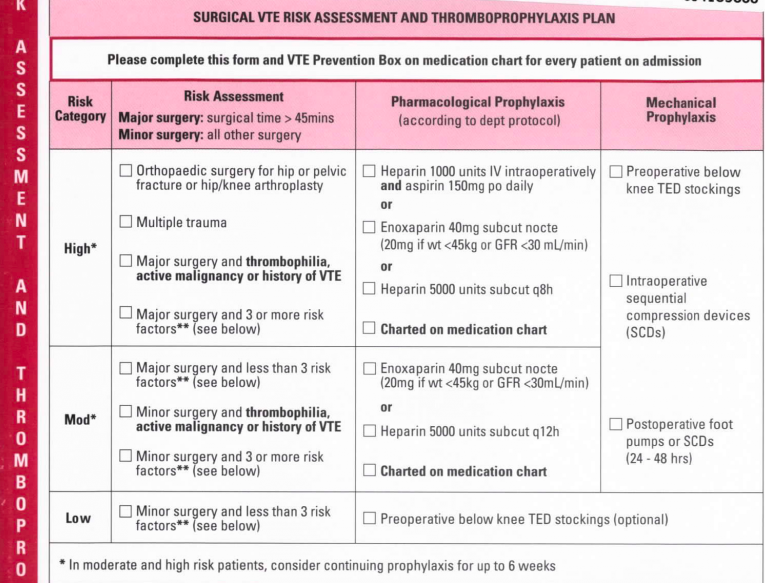


**
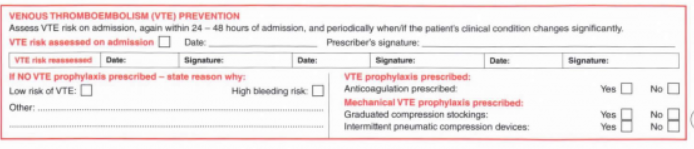
**

# Supplementary file 4. Informal VTE risk assessment checklist for orthopaedics

**
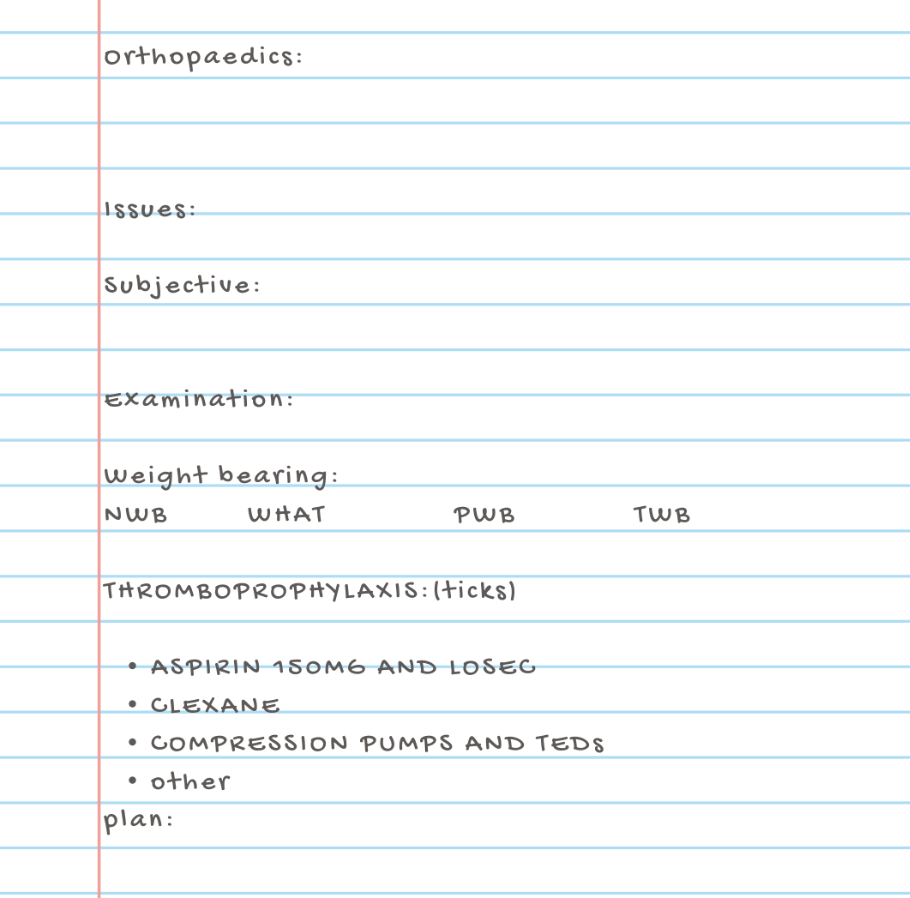
**
